# Supplementary material for: Perception of biological motion by jumping spiders
Source: PLoS Biol. 2021 Jul 15;19(7):e3001172. doi: 10.1371/journal.pbio.3001172 (PMC8282030; doi:10.1371/journal.pbio.3001172)
Supplement: Attachment — Submitted filename: Answer_to_Reviewers.pdf [file pbio.3001172.s015.pdf]

Reviewer #1:

The paper reports the first evidence of recognition of biological motion in an invertebrate species, a finding that I believe deserves to be published. I have only minor requests for changes.
MDA) We thank the reviewer for the work that they contributed to this review. Please see the response to all your comments below.

p. 2 second para. line 10: This is incorrect of course. Human newborns cannot have been completely prevented the possibility to see motion patterns. Direct evidence is possible only in controlled conditions in animal models (e.g. the Plos Biology 2005 paper quoted below on newly-hatched chicks does provide such an evidence). This part should be better formulated. Please also consider on the same topic Gravity bias in the interpretation of biological motion by inexperienced chicks. Current Biology (2006), 16: 279-280.
MDA) Indeed, we agree with the reviewer: human infants' studies suffer from the impossibility of controlling previous experiences. We have now reduced the claim about human infants and moved the innateness statement to the animal models. (L57-59)

p. 2 second para. line 15: On the 'animacy detector' see also Vallortigara, G. (2012). Aristotle and the chicken: Animacy and the origins of beliefs. In "The Theory of Evolution and its Impact" (A. Fasolo, ed.), pp. 189-200, Springer, New York.
MDA) We have added the suggested reference, thank you.

p. 3 second para. the part starting from "Neuroanatomically..." This seems to be of little importance with respect to the rest of the paper and should be reduced or better omitted altogether.
MDA) The reference to the neuroanatomical structure of the visual system was intended as a further suggestion that the secondary eyes may be responsible for visual processing and discrimination. We have now moved this section slightly, and shortened it, to make this reference more clear, thank you. (L95-101)
p. 6 line 5 and line 11: change "agent detection" with "animacy detection" (recognition of agency is different than recognition of 'animacy')
MDA) Corrected, thank you.

p. 6 second para., the last three lines "How this might be implemented..." should be omitted.
MDA) This has been removed, thank you.

p. 7 line 2: "animacy detection" not agent
MDA) This has been removed, thank you.

Reviewer #2:

This manuscript details the responses of salticid spiders toward point light stimuli with different degrees of biological relevance (or 'realism') with respect to the movement depicted by the dots. This is a novel and interesting method and investigative tool to use on this group of animals. The results were somewhat surprising, and the authors have done a reasonable job of interpreting these results. I have mostly fairly minor comments, although there are some fairly glaring mistakes with the use of terminology of the eyes, and with respect to the descriptions of the fields of view (fov) of the different pairs of eyes. These can be easily rectified.
MDA) We thank the reviewer for their work in reviewing our manuscript. We are also happy that the methodology was well received regarding its potential for future studies. Regarding the reviewer's comments, please see the comments in line below.

My principal comment is that, as you tested male and female spiders, and the sexes are known to be somewhat different in their contrast (and other) visual thresholds (Zurek work), it would have been interesting to see a comparison between males and females in this piece of work. I know this would require additional analysis, but I do think that this would make the current manuscript considerably stronger, both in content, and in its ability to make predictions about the visual system of this group of animals. Given that you have the rotational paths of each individual, and you know M/F, it should not be too hard?
MDA) Although we chose not to include this analysis in the main text of the original version of the manuscript, we agree with the reviewer that especially for scientists interested in spider behavior a comparison between males and females would be informative. We have therefore added two new GLMMs and associated analysis to the supplement. In the first of these newly added models, sex was added as a predictor to the directional peak magnitude. This model provides insight into how the different sexes respond to the different stimuli in each condition. In the second, we included sex as a predictor to the absolute peak magnitude. This model provides insight on the overall activity in the different sexes (independent from which of the two stimuli in the pair is preferred). Overall, the results of both models are consistent with the conclusions presented in the main text.

As these models are more complex, and as such their statistical power is lower, we do not want to overstate or over-interpret their results and would prefer leaving them in the supplement. (However, if the reviewer and the editor feel strongly that they deserve a position in the main text, we would be willing to include them there.)

We report here the findings for your convenience:

1. directional magnitude

```
## Analysis of Deviance Table (Type II Wald chisquare tests)
##
## Response: dirval_deg_s
##                             Chisq Df Pr(>Chisq)
## stimn                      0.8094  1  0.3682947
## cond                       3.1825  3  0.3643392
## stimpres                  74.4291  1  < 2.2e-16 ***
## sex                        3.0909  2  0.2132198
## stimn:cond                 2.5505  3  0.4662277
## stimn:stimpres             2.2476  1  0.1338183
## cond:stimpres             18.9353  3  0.0002819 ***
## stimn:sex                  0.0817  2  0.9599919
## cond:sex                   7.4838  6  0.2784137
## stimpres:sex               1.4923  2  0.4741895
## stimn:cond:stimpres        2.2741  3  0.5175047
```

```
## stimn:cond:sex            7.1002  6  0.3116801
## stimn:stimpres:sex        2.9268  2  0.2314461
## cond:stimpres:sex        15.8582  6  0.0145358 *
## stimn:cond:stimpres:sex   8.5662  6  0.1994853
## ---
## Signif. codes:  0 '***' 0.001 '**' 0.01 '*' 0.05 '.' 0.1 ' ' 1
```

As shown here, even when adding sex as a predictor, our main effects of stimulus presence and its interaction with conditions remains, as reported in the results shown in the main text.

Regarding sex, there seems to be no main effect, meaning that females, males and juveniles all respond with overall similar frequency. Moreover, no two-ways interaction with sex result significant. There is instead an effect of the interaction between condition, stimulus presence and sex, probably meaning that for some sexes, in some condition, the difference in turning likelihood during the stimulus presentation and inter-stimulus time changes.

We will proceed with a post hoc analysis, to observe the directionality of such differences

```
##  cond         stimpres sex  emmean    SE    df t.ratio p.value
##  bio-rand     0        f     1.181  1.96 20947   0.603  1.0000
##  bio-scram    0        f     4.146  1.98 20947   2.089  0.5924
##  scram-rand   0        f     2.878  1.95 20947   1.473  0.9738
##  shil-ellipse 0        f     5.030  2.20 20947   2.285  0.4180
##  bio-rand     1        f   -14.364  4.18 20947  -3.434  0.0142
##  bio-scram    1        f     0.647  4.19 20947   0.154  1.0000
##  scram-rand   1        f   -16.221  3.94 20947  -4.119  0.0009
##  shil-ellipse 1        f   -25.942  4.25 20947  -6.109  <.0001
##  bio-rand     0        j     4.096  3.72 20947   1.101  0.9995
##  bio-scram    0        j    -1.234  3.23 20947  -0.382  1.0000
##  scram-rand   0        j     3.281  5.56 20947   0.590  1.0000
##  shil-ellipse 0        j    -1.143  4.24 20947  -0.269  1.0000
##  bio-rand     1        j   -21.984  7.48 20947  -2.940  0.0760
##  bio-scram    1        j     1.000  7.10 20947   0.141  1.0000
##  scram-rand   1        j     2.418 10.45 20947   0.231  1.0000
##  shil-ellipse 1        j   -28.418  7.98 20947  -3.563  0.0088
##  bio-rand     0        m    -4.615  3.93 20947  -1.173  0.9987
##  bio-rigid    0        m    -2.389  3.67 20947  -0.650  1.0000
##  scram-rand   0        m     0.479  4.05 20947   0.118  1.0000
##  shil-ellipse 0        m     2.538  3.71 20947   0.684  1.0000
##  bio-rand     1        m    -3.308  8.49 20947  -0.390  1.0000
##  bio-scram    1        m   -13.029  7.40 20947  -1.761  0.8585
##  scram-rand   1        m   -30.865  8.57 20947  -3.602  0.0076
##  shil-ellipse 1        m    -9.362  7.79 20947  -1.202  0.9981
##
## P value adjustment: sidak method for 24 tests
```

- As expected, in all the comparisons regarding the inter-stimulus time (when no stimulus was present onto the screen) every group shows no difference from chance level. Moreover, in all the three sex groups, there is no statistically significant preference for either stimulus in the biological vs scrambled condition.
- Regarding female spiders, the observed result is identical to the one observed in the full model: preference for the random stimulus in the random vs scrambled and random vs biological conditions; preference for the ellipse in the ellipse vs silhouette condition.
- For male spiders, only the scrambled vs random condition maintain significance, while the other three result at chance level. As stated above, we are a bit wary about giving a

direct interpretation since we are looking at only 10 subjects. However, the direction of preference seems consistent with the main experiment, with even a surprisingly similar direction in the biological vs scrambled condition. Indeed, the biological stimulus looks like a spider, while the scrambled one even though perceived as alive, does not. It is possible that males jumping spiders are more specific in their preference: rather than distinguishing living from non-living things, it may concentrate on spiders vs non-spiders. Further experiments may look into such discrimination

- For juveniles, we still see an effect in the silhouette vs ellipse condition, along with a borderline significance in the biological vs random condition. On the other hand, there seems to be no preference in the biological vs scrambled and the scrambled vs random conditions. While reiterating our advised caution in the interpretation, it is possible that younger, inexperienced spiders perform worse in this discrimination task, suggesting a partial dependence from experience (or sexual maturity). As such, easy comparisons (random vs biological and silhouette vs ellipse) are performed correctly, while comparisons with the scrambled stimulus result more confounding.

2. absolute magnitude

```
## Analysis of Deviance Table (Type II Wald chisquare tests)
##
## Response: absval_deg_s
##            Chisq Df Pr(>Chisq)
## sex       7.0355  2    0.02967 *
## cond      2.3430  3    0.50434
## sex:cond 1.2079  6    0.97649
## ---
## Signif. codes:  0 '***' 0.001 '**' 0.01 '*' 0.05 '.' 0.1 ' ' 1
```

There seems indeed to be an effect of sex, but no interaction with condition. Performing a post-hoc analysis:

```
##  contrast estimate     SE    df t.ratio p.value
##  f - j      0.1757 0.0665 20983   2.642  0.0225
##  f - m      0.0681 0.0799 20983   0.853  0.6701
##  j - m     -0.1075 0.0891 20983  -1.207  0.4490
##
## Results are averaged over the levels of: cond
## Results are given on the log (not the response) scale.
## P value adjustment: tukey method for comparing a family of 3 estimates
```

females respond with a higher rate over juveniles. The estimate value is very similar also in the juveniles vs males contrast, but probably given the low number of subjects in these two groups it does not rise to significance. Curiously, on the contrary of what found in the literature, female and males seem to respond at a similar rate.

It is worth pointing out that most target detection studies in jumping spiders tend to present a prey-like stimuli. In such conditions, the animal motivation and hunger are big factors in determining the attention level, with females generally eating more than maes. In this experiment instead, many of the stimuli depict (or should resemble) conspecifics, the detection of which is not bound to the same foraging motivation.

All other comments and notation is made with respect to pages on downloaded pdf, as there are no line numbers.

On page 9, I am unclear how in joints "relative distances varying across time,". Do you mean phylogeny? I think you don't mean that - perhaps delete "across time", or of not clarify what you mean.
MDA) With "across time" we wanted to refer to the dynamics of the motion. For example while walking, the distance between two different joints (two wrist for example) varies across time. We agree that the sentence as we put it can be misleading. This have been corrected, thank you.(L45)

P10. Eye structure- this is incorrect. Note that the PME have a very narrow field of view, if any at all, in most species (where this pair of eyes is typically vestigial). The ALE and PLE have wide fields of view. See Land, M. F. (1985a). "Fields of view of the eyes of primitive jumping spiders." J. exp. Biol. 119: 381-384
MDA) We are sorry about the confusion and thank the reviewer for this note. We have corrected this point in the text. (L86-89)

Fig 2C. I am having some trouble working out the dark lines (mean) versus shaded area (SEM) - partly because there is coloured shading representing stimulus ON/OFF screen. Can a dashed line be used for the mean and shading for the SEM?
MDA) We have now clarified the figure, thank you. Specifically, now in figure 2C the means are represented with dashed lines, while the shaded area represent the SE. Moreover, we added a legend on top for the inter stimulus section and the during stimulus presentation respectively. Lastly, we changed the stimulus position line in figure 2B, making it dashed.

End of results. For people who understand salticid eyes and the set-up, it is not especially surprising that stimuli elicited a response at about 4.5 s, when they were within the fov of the ALE. However, for others, this sentence might appear a bit 'random'. I would make this clear.
MDA) We have added the specification about the ALE field of view in the text, thank you. We remained cautious in the text, as we don't really have a direct information of the fov in *Menemerus*, which may be slightly different from *Plexippus* (Land 1985) or *Servea* (Zurek & Nelson 2012). (L172-174)

P 12. "detection of a target with the lateral eyes" - you should be consistent. This is not technically correct. You should be referring here to the secondary eyes unless you are discussing solely your results in the narrower context, in which case this should be made clear and it should be stated that this is the ALE you are referring to and specifically with respect to this experiment.
MDA) Thank you for this point, and the reviewer is correct. W now use "secondary eyes" in the text to make this more clear.

P. 13, beginning. "…AME on the stimulus which cannot be decoded with the lateral eyes alone, particularly since the other target will still remain in the visual field of the secondary eyes following rotation." The work in Zurek, D. B., et al. (2010). "The role of the anterior lateral eyes in the vision-based behaviour of jumping spiders." Journal of Experimental Biology 213(14): 2372-2378, seems especially pertinent here, as it clearly demonstrated the spatial ability of the ALE, which is something that the authors do not seem to be aware of. This reference is also pertinent to your later statement: "That spiders demonstrate this preference even when targets can only be viewed by the secondary eyes is striking". The point made later, about discriminations being made on motion is very valid, and an interesting one.
MDA) We thank the reviewer for this point, and we fully agree—particularly regarding the section where we discuss the discrimination abilities of the AME and ALE, where directly highlighting the visual acuity of the ALE would be helpful to the reader. We have now extended

that section slightly and added the suggested reference. For your convenience, we have included that corrected section here:

> **L217-226:** That spiders demonstrate this preference even when targets can only be viewed by the secondary eyes is striking. Indeed, multiple studies have been carried out exploring the visual discrimination abilities of jumping spiders (De Agrò, 2020; Dolev and Nelson, 2016, 2014), and the secondary eyes have been found to possess high spatial acuity (Jakob et al., 2018; Zurek et al., 2010; Zurek and Nelson, 2012a). However, although it has been suggested that these eyes are capable of feats beyond mere motion detection (Spano et al., 2012), our results demonstrate that these eyes can solve a discrimination task without aid from the primary eyes. Further, as point-light displays are designed to contain minimal visual detail, the discrimination operated by the secondary eyes must be based on motion.

P. 13. Some salticid species are, in fact, somewhat social. I would add the caveat "jumping spiders are not TYPICALLY social"
MDA) This have been corrected, thank you.

Very minor

Sometimes "cm" is written directly after the number (no space), and other times there is a space.
MDA) This have been corrected, thank you.

Reviewer #3:

In this interesting research paper, De Agrò and colleagues investigate the responses of jumping spiders to visual displays presented on a computer screen. The Authors focus on response to biological motion vs other manipulations of visual stimuli implemented using point light displays and silhouettes. Similarly to other studies conducted in vertebrate species, the Authors used pairs of stimuli including point displays of biological motion (that move semi-rigidly following the movement of a spider) vs scrambled motion vs random motion. They also tested a silhouette of a spider vs an ellipse. All stimuli translated horizontally, while joints/silhouettes were manipulated in different ways.
The results solidly support the idea that in jumping spiders biological motion produces different behavioural responses (eg saccades) compared to random motion and scrambled motion.
Although the visual representation of results is less than straightforward, the results appear robust and novel.
MDA) We thank the reviewer for the suggestions. We are happy to see that our manuscript was well received. Please see below for responses to each of your comments.

It would be interesting to know whether all spiders had similar responses or whether the effect is driven by few individuals.
MDA) Every subject saw 10 stimulus pairs per condition, which is unfortunately not enough to draw any definitive conclusions about individual differences. As per another reviewer's question however, we have added an analysis about sex differences in supplement 1. We still think that the number of observations per sex group mean that these results should be interpreted with caution, but these results may be useful to the interested reader, nonetheless.

Can the Authors clarify how many saccades were present in each condition?
MDA) Our analysis focused on changes in rotational position, rather than counting individual saccades. Although this method is a bit less intuitive, it provides a number of crucial advantages. For example, were we to use number of observed Z-peaks per stimulus, per direction, this would have overestimated the effect of random noise. Conversely, had we used a strict magnitude-based threshold we would have risked excluding smaller rotations—a particularly problematic challenge as rotations would be expected to get smaller as the stimulus nears the center of the screen (and thus directly in front of the spider). As such, we cannot provide saccades frequency without being forced to operate one of these choices, which would be completely subjective. For these reasons, we chose a method with minimal computation (compared to a score of some sort), which also allowed us to maximize the effectiveness of the GLMM, which can filter out the noise by itself—resulting in a process that yielded extremely reliable analyses and which also minimized the decisions imposed by the experimentalists.

The Authors commented on the "unexpected" result of a preference of spiders for orienting towards the less naturalistic stimulus. Some potential interpretations have not been mentioned. For instance: Can this be due to the fact that the more naturalistic stimuli are considered as potential competitors for catching a potential prey? Is it possible that more realistic stimuli are considered potential predators to flee away from? These two options are basically prey capture and predator avoidance or competitor avoidance. Other solitary species appear to actively move away from conspecifics (e.g. tortoise hatchlings), so there are several possible reasons behind this outcome.
This study leads to further questions on whether the size of objects and type of biological motion can influence the direction of preference.
MDA) We thank the reviewer for the great insight. In our paper, we do suggest an explanation for this reversed preference:

> **L189-196:** As described above, these animals produce saccades upon detection of a target with the secondary eyes, allowing further inspection with the AME. As per our initial hypothesis, the secondary eyes may be immediately able to decode motion-based information, enabling them to determine which of the stimuli requires more detailed investigation. In a forced-choice paradigm it may be advantageous to focus the AME on the stimulus which cannot be decoded with the secondary eyes alone, particularly since the other target will still remain in the visual field of the secondary eyes following rotation."

The proposed hypotheses are instead more bound to the animal ecology. Indeed, the stimuli we presented were bigger than the subjects, being around 4cm in length, which may have caused it to be perceived as a competitor or predator. However, we think that this is an unlikely explanation for the observed behaviours. If the biological stimulus was indeed perceived as a predator, we would have expected for the spider to turn towards it and then run away, rather than turning its attention away from it.
A brief reference to this concept has been added to the main text. We report it below for your convenience:

> **L183-188:** Even though the stimuli may have been perceived as predators or competitors, given their size, it seems unlikely that our result can be explained as an avoidance effect. Rather than turning towards the "less dangerous" stimulus under this perspective, we would have expected spiders to have maintained their attention on the "more dangerous" stimulus, and possibly attempted to run away.

I would like to ask the Authors to consider to use a more straightforward visual representation to help the reader. For instance, can they use legends for the different coloured lines? Is it possible make a connection between the unit of measurement to the variable of interest? (e.g. rotation toward the object?).
MDA) Thank you for this suggestion, we have now improved the readability of the graph. Specifically, now in figure 2C the means are represented with dashed lines, while the shaded area represent the SE. We also added a legend indicating the inter stimulus section and the stimulus presentation section, respectively. Lastly, we changed the stimulus position line in figure 2B, making it dashed to improve readability. We felt that a legend would be unreadable here, although having the line as dashed should clarify the figure caption. Regarding frequency of rotation, please see the response provided above concerning our decision to use delta z-axis rotation.

In the Data analyses part I was initially confused by the X vs Y axis of the sphere. Would it be worth to add a visual representation of the sphere and its axis?
MDA) We have now added a small scheme of the sphere in Figure 2A, and specified the direction of the saccades in figure 1A, thank you.

This paper would also benefit from a video of the subjects performing the task.
MDA) We have now added to the supplements a brief video of a spider on the sphere during a trial, thank you.
