# Supplementary material for: Perception of biological motion by jumping spiders
Source: PLoS Biol. 2021 Jul 15;19(7):e3001172. doi: 10.1371/journal.pbio.3001172 (PMC8282030; doi:10.1371/journal.pbio.3001172)
Supplement: Attachment — Submitted filename: Answer_to_Editor.odt [file pbio.3001172.s016.odt]

Dear editor,

We thank you very much for your work and for considering our paper for publication. As requested, we:

Changed the title to the suggested one. We have updated the title both in the manuscript file and the manuscript metadata.
Wrote a short blurb to accompany the article. You can find it in the manuscript file first-page.
Re-uploaded the raw data file with the name SI1_Data.xlsx. For consistency, we also re-uploaded the zip file with the full analysis and the python script as SI2. We also added in the description of figure 2 a reference to the raw data file. We specified that the data for panel A and B come directly from SI1, while panel C report average and confidence intervals calculated with the GLMM reported in SI2, based on the data in SI1. We did not add the same reference in figure 1, as it’s only descriptive (of the apparatus and the stimuli) and as such not based on the collected data.

Best regards,
Massimo De Agrò, on behalf of the co-authors.
